# The ICET-A Recommendations for the Diagnosis and Management of Disturbances of Glucose Homeostasis in Thalassemia Major Patients

**DOI:** 10.4084/MJHID.2016.058

**Published:** 2016-10-28

**Authors:** Vincenzo De Sanctis, Ashraf T. Soliman, Heba Elsedfy, Saif AL Yaarubi, Nicos Skordis, Doaa Khater, Mohamed El Kholy, Iva Stoeva, Bernadette Fiscina, Michael Angastiniotis, Shahina Daar, Christos Kattamis

**Affiliations:** 1 Adolescent Outpatient Clinic, Quisisana Hospital, Ferrara, Italy; 2 Department of Pediatrics, Division of Endocrinology, Alexandria University Children’s Hospital, Alexandria, Egypt; 3 Department of Pediatrics, Ain Shams University, Cairo, Egypt; 4 Pediatric Endocrine Unit, Department of Child Health, Sultan Qaboos University Hospital, Al-Khoud, Sultanate of Oman; 5 Division of Pediatric and Adolescent Endocrinology, Paedi Center for Specialized Pediatrics, St. George’s University Medical School at the University of Nicosia, Cyprus; 6 Department of Pediatrics, Endocrinology Unit, Alexandria University Children’s Hospital, Egypt, and Child Health Department, Sultan Qaboos University Hospital, Muscat, Oman; 7 Paediatric Endocrinologist,”Screening and Functional Endocrine Diagnostics” SBALDB. Professor Ivan Mitev, Medical University Sofia, Bulgaria; 8 Department of Pediatrics, NYU School of Medicine, New York, USA; 9 Medical Advisor, Thalassemia International Federation (TIF), Nicosia, Cyprus; 10 Department of Hematology, College of Medicine and Health Sciences Sultan Qaboos University Oman, Sultanate of Oman & Visiting Scholar, Stellenbosch Institute for Advanced Study (STIAS), Wallenberg Research Centre at Stellenbosch University, Stellenbosch 7600, South Africa; 11 First Department of Paediatrics, University of Athens, Athens, Greece

## Abstract

Iron overload in patients with thalassemia major (TM) affects glucose regulation and is mediated by several mechanisms. The pathogenesis of glycaemic abnormalities in TM is complex and multifactorial. It has been predominantly attributed to a combination of reduced insulin secretory capacity and insulin resistance. The exact mechanisms responsible for progression from norm glycaemia to overt diabetes in these patients are still poorly understood but are attributed mainly to insulin deficiency resulting from the toxic effects of iron deposited in the pancreas and insulin resistance. A group of endocrinologists, haematologists and paediatricians, members of the International Network of Clinicians for Endocrinopathies in Thalassemia and Adolescence Medicine (ICET-A) convened to formulate recommendations for the diagnosis and management of abnormalities of glucose homeostasis in thalassemia major patients on the basis of available evidence from clinical and laboratory data and consensus practice. The results of their work and discussions are described in this article.

## Introduction

β Thalassemias are a group of inherited chronic hemolytic anemias characterized by reduced (β ^+^) or absent (β ^0^) synthesis of the β globin chains of the haemoglobin A tetramer. They are particularly common in people of Mediterranean, African, and Southeast Asian ancestry. More than 30,000 babies are born with homozygous β-thalassaemia worldwide each year and there are 100 million individuals who are asymptomatic β-thalassaemia carriers. Three clinical and haematological conditions of increasing severity are recognized: the β-thalassemia carrier state, β-thalassemia intermedia (Non Transfusion Dependent Thalassemia; NTDT) and β-thalassaemia major (TM).^1^,^2^

Today, nearly all subjects with TM survive into adult life, and many patients who have access to excellent care with proper chelation survive beyond 50 years of age. The improved survival of patients with TM results in an increasing prevalence of complications of iron overload, including abnormalities of glucose homeostasis. Disturbances of glucose homeostasis range from increased insulin resistance and mild glucose intolerance to overt diabetes mellitus. Patients with mild disorders are usually asymptomatic; impaired glucose tolerance (IGT) is common, occurring in up to 24.1%.^3^–^5^ Unfortunately, this represents an additional potential risk to their cardiac function.^6^

Although iron overload induced DM shares certain characteristics with both type 1 diabetes and type 2 diabetes, it appears to be a separate entity with a unique pathophysiology. As in type 1 DM, insulin deficiency is a primary defect; however, it is usually relative rather than absolute. Similar to type 2 DM, the onset of the disease is usually gradual and insidious and insulin resistance is detected in some patients.^3^,^4^

Therefore, patients with TM and health professionals should be aware of the high incidence of glucose abnormalities in patients with thalassemia syndromes.^5^,^7^ Detecting the pre-diabetes stage is critical because prediabetes and clinical diabetes can potentially be reversed or prevented with optimum chelation treatment.^5^,^7^

## Aims

The International Network of Clinicians for Endocrinopathies in Thalassemia and Adolescent Medicine (ICET-A)^8^ planned the current project to formulate recommendations for accurate diagnosis and effective management of abnormalities of glucose homeostasis in patients with TM.

A brief review and description of the clinical management of these patients is also provided with particular attention to the assessment, prevention, and treatment of iron overload.

The aim of this project is to support the clinical practice of paediatricians, internists, haematologists and other physicians who care for patients with TM.

## Diagnostic Criteria Used for the Assessment of Glucose Abnormalities

The diagnosis of IGT and DM is currently made during a period of stable baseline health according to standard American Diabetes Association (ADA criteria).^9^

– **Criteria for diagnosis of diabetes mellitus (DM):**
  ○ With classic symptoms of hyperglycemia or hyperglycemic crisis; a random plasma glucose ≥ 11.1 mmol/L (≥ 200 mg/dL).
  ○ Fasting plasma glucose (FPG) ≥ 7.0 mmol/l (≥ 126 mg/dl) or 2-hour plasma glucose ≥ 11.1 mmol/l (≥ 200 mg/dl). Fasting is defined as no caloric intake for at least 8 h.
  ○ Hemoglobin A1c (HbA1c) ≥ 6.5%.

The test should be performed in a laboratory using a method that is National Glycohemoglobin Standardization Program (NGSP) certified and standardized to the Diabetes Control and Complications Trial (DCCT) assay.

- **Criteria for increased risk for diabetes (prediabetes):**
  ○ Fasting plasma glucose (FPG) between 100 mg/di (5.6 mmol/L) to 125 mg/dl (6.9 mmol/l).
  ○ 2-h PG, in the 75-g OGTT, between 140 mg/dl (7.8 mmol/L) to 199 mg/dL (11.0 mmol/L). The test should be performed as described by the WHO, using a glucose load containing the equivalent of 75 g anhydrous glucose dissolved in water. In the absence of unequivocal hyperglycemia, results should be confirmed by repeat testing.
  ○ HbA1c between 5.7 and 6.4%

The Canadian Diabetes Association Clinical Practice Guidelines Expert Committee^10^ recommends that the decision of which test to use for diabetes diagnosis is left to the clinician’s judgement. Each diagnostic test has advantages and disadvantages. In the absence of symptomatic hyperglycemia, if the result of a single laboratory test is in the diabetes range, a repeat confirmatory test must be done on another day. It is preferable that the same test is repeated (in a timely fashion) for confirmation.

In the case of symptomatic hyperglycemia, the diagnosis has been made, and a confirmatory test is not required before treatment is initiated. If results of 2 different tests are available and both are above the diagnostic cut-off points, the diagnosis of diabetes is confirmed. When the results of more than one test are available, and the results are discordant, the test whose result is abnormal should be repeated and the diagnosis made on the basis of the repeat test.^10^

## Formulation of Recommendations for the Diagnosis and Management of Disturbances of Glucose Homeostasis in Thalassemia Major Patients

A systematic search of PubMed and Google Scholar from May 2006 through September 2016 was performed. Searches were prospectively limited to publications in the English language. MeSH terms and strings used in various combinations in the literature search included: thalassemia combined with diabetes (507 references), impaired glucose tolerance (83 references), anemia (380 references), iron overload (144 references), chronic liver disease (352 references), chelation therapy (94 references), zinc (204 references), treatment of diabetes (34 references), diabetes and complications (264 references). Recommendations from published guidelines were also used when available and appropriate (11 references).

## Organization and Evidence Levels

Two chairmen (VDS and ATS) appointed an expert panel of pediatricians, endocrinologists, and haematologists, selected for their expertise in research and the clinical treatment of thalassemia. This advisory committee, chaired by nine clinicians to support the systematic review of the literature and to guarantee the accuracy of the process, suggested the use of a unified system called the Strength of Recommendation Taxonomy (SORT) developed by editors of the US family medicine and primary care journals (ie, American Family Physician, Family Medicine, Journal of Family Practice, and BMJ USA).^11^–^14^ Evidence was graded using a 3-point scale based on the quality of methodology (e.g., randomized control trial, case control, prospective/retrospective cohort, case series, etc.) and the overall focus of the study as follows:

- Good-quality patient-oriented
- Limited-quality patient-oriented evidence
- Other evidence, including consensus guidelines, opinion, case studies, or disease oriented evidence.

The strength of recommendation was ranked as follows:

- Recommendation based on consistent and good-quality patient-oriented evidence.
- Recommendation based on inconsistent or limited-quality patient-oriented evidence.
- Recommendation based on consensus, opinion, case studies, or disease-oriented evidence.

## Process Followed for the Preparation of Manuscript

The two chairmen, pediatric endocrinologists (VDS and ATS), prepared the draft article which was subjected to scrutiny by a panel of experts consisting of six pediatric endocrinologists (HE, SA, NS, DK, MEK and IS), one paediatrician (BF), and three pediatricians/thalassemiologists (MA, SD and CK) with at least four decades of experience in this field. During the preparation of the draft, comments from members of the ICET-A Network (hematologists, thalassemiologists, paediatricians, and endocrinologists) were also considered. Final clinical recommendations and observations were prepared by the Steering Committee and approved by the ICET-A Network Board for use by any healthcare professional managing TM patients. The interpretation and application of clinical practice recommendations will remain the responsibility of the individual clinician. The recommendations will be considered current for a period of 3 years from the date of publication unless reaffirmed or updated before that time.

Due to the large number of references reported in the literature, the Steering Committee decided to cite only the scientific publications on which our work is based.

In those situations where documented evidence based data were not available or were showing inconsistent or limited conclusions, expert ICET-A opinion and the medical consensus was used to generate clinical recommendations.

## Management of β-Thalassemia Major

### a. Transfusions

Clinical management of TM consists of regular life-long red blood cell transfusions (RBCs) and iron chelation therapy to remove the excess transfusional iron. Current guidelines for the treatment of anemia in TM recommend transfusions at a hemoglobin (Hb) level of more than 9.0% g/dl, which is associated with adequate inhibition of bone marrow expansion. In patients with TM, the rate of transfusional iron loading should be monitored and considered when choosing the appropriate dose of an iron-chelating agent.^15^

### b. Iron overload and its management

The characteristic pattern of iron deposition with regular transfusions initially involves iron storage as ferritin and hemosiderin in reticuloendothelial cells such as macrophages of the spleen, liver, and bone marrow. This is followed by iron accumulation elsewhere, mainly in hepatocytes, but also in endocrine glands, anterior pituitary, and myocardium.

Three iron chelator drugs are currently approved: deferoxamine (DFO), deferiprone (DFP) and deferasirox (DFX). Iron excretion induced by chelators is the sum of urinary and faecal iron excretions. For deferoxamine, urinary iron excretion represents around 50% of the total, for deferiprone 80–98% and for deferasirox less than 5%.^16^–^23^

A variety of factors differentiate the currently available iron chelators, including the mode of administration, the dosing schedule, the chelator’s ability to remove iron from different organs (i.e., heart, liver) and the adverse effects. These factors should be carefully considered when choosing a chelation regimen.^18^

The availability of more than one iron chelating drug stimulated the studies for benefits from combination therapy. Combination treatment (two drugs daily taken at full doses and simultaneously or alternating the two drugs during the week) may be considered every time there is a need to look for an additive or synergistic effect in patients with severe iron overload and heart disease.^22^,^23^ A single uncontrolled study suggests that combination therapy (DFO plus DFP) may reverse endocrine complications such as glucose intolerance in patients with TM.^24^ The same holds true with optimum monotherapy preserving low iron load and iron negative balance.

## Assessment of iron overload

The accurate evaluation of iron overload is crucial in order to plan and monitor iron chelation therapy. Multiple methods of assessing the degree of iron overload exist and each method has benefits and limitations. In clinical practice, combinations of the different techniques and serial measurements are used to assess the iron burden and to adjust chelation therapy.^25^,^26^ Invasive methods include liver and heart biopsies. In general, ubiquitous access to non-invasive methods has replaced biopsies as the standard method for measuring tissue iron concentrations in most centres. The non-invasive methods of measuring iron overload include serum ferritin (SF), non-transferrin bound iron (NTBI), labile plasma iron (LPI) and liver iron concentration (LIC) as determined by MRI R2*, liver superconducting quantum interference device (SQUID) and cardiac T2* MRI.^27^

### a. Serum ferritin (SF)

In the majority of clinical centres, the standard method of evaluating the total amount of body iron is a measurement of SF concentration in the blood.

In the absence of confounding factors, such as inflammation, vitamin C deficiency, oxidative stress, liver dysfunction and increased cell death, SF is proportional to the degree of cellular iron stores. Therefore, serial assessments are recommended.^2^

### b. Liver iron concentration (LIC)

The liver contains most of the body iron stores (70–80%) and is the main crossroads of iron trafficking (storage from intestinal absorption and from red-cell catabolism, chelation by iron chelating drugs and excretion through bile). Several studies have linked very high LIC (> 15 mg/g dry weight) to worsening prognosis, liver fibrosis progression and hepatocellular carcinoma. Levels above 7 mg/g dry weight are indications to increase chelation since a major risk of complications occur at levels 7–14.^28^

LIC can also be measured accurately using SQUID and MRI. SQUID has been validated but has limited availability and cannot measure iron in the heart. MRI is widely available, robust and reproducible. Inter-observer variability is insignificant and inter-study variability is approximately 5%–7%. Variability among scanners is also small.^29^,^30^

### c. Iron load of other tissues and organs and MRI

The introduction of MRI for the assessment of tissue iron in the early 2000s completely changed our understanding of iron overload and its management. This method is non-invasive, cost-effective, with no radiation exposure, and of widespread availability.^31^–^40^

MRI has proven effective in detecting and accurately quantifying iron in the heart, liver and other organs, including endocrine glands (pancreas, pituitary and adrenal). Pituitary R2 correlated significantly with serum ferritin as well as liver, pancreatic, and cardiac iron deposition.^34^–^36^ One significant advantage of cardiac MRI is its ability to recognize preclinical cardiac iron deposition, allowing effective early treatment and so preventing progression to heart failure.

T2* is the time needed for the organ to lose approximately two-thirds of its signal and is measured in milliseconds (ms). T2* shortens as iron concentration increases. Its reciprocal, 1000/T2*, is known as R2* and is measured in units of inverse seconds (S^−1^).

However, pancreas R2* measurements have several limitations: (a) they have not gained widespread use, (b) functional correlates require further investigation and (c) the pancreas may be difficult to locate in older, splenectomized thalassemia major patients because of glandular apoptosis, fatty replacement, and loss of normal anatomic landmarks.^37^–^39^

#### Recommendations

- Current practice is to start chelation therapy after transfusion of 5–10 units of blood (approximately 1–2 gr/Fe), or when the ferritin level rises above 1,000 μg/l. (I,C)
- Serum ferritin has been used to start, formulate and monitor chelation therapy, but it is now known to be an imprecise indicator of total body iron burden since it can yield inappropriate results in the presence of inflammation, abnormal liver function or ascorbate deficiency. Despite these reservations, trends in serum ferritin concentrations serve as a reasonable, cost efficient and readily applicable surrogate marker for the iron load. (I,C)
- LIC estimation using MRI shows excellent correlation with that obtained from liver biopsy and is an accurate method to assess liver iron content and proportional iron stores.(I,A)
- Pancreatic imaging has a potential role in the assessment of iron deposition and for the prediction of the development of glycemic abnormalities. (I,B)
- Prospective data are needed to prove the validity of pancreatic MRI imaging for the assessment of effects of different chelators as well as their doses; more evaluation is required before this measurement can be recommended for routine use. (II,C)

## Prevalence of Glucose Abnormalities in Patients with TM

Glucose tolerance abnormalities and DM are common complications in patients with TM.

Pancreatic iron loading in these patients begins after the first decade of life and the incidence of complications increases with age. The rate of iron accumulation is directly related to the annual blood consumption, the delay in starting chelation and to low compliance and/or inadequate chelator doses. While glucose intolerance occurs at an early stage of adolescence, DM frequently occurs at later stages and is usually secondary to iron overload and subsequent chronic liver disease.

Depending on the age composition of cohorts, up to 25% of patients with TM may have isolated impaired fasting plasma glucose (FPG), a condition in which the fasting blood glucose is elevated above what is considered normal, but is not high enough to be classified as DM.^39^–^41^ FPG has a good correlation with other glycemic indices such as fasting insulin, insulin resistance index and beta cell function index. Impaired FPG is considered a pre-diabetic state. However, it is not known how many patients with TM with impaired FPG progress over the years to diabetes.^41^

The prevalence of DM and IGT in adolescents and young adults with TM conventionally treated with DFO varies in different series (up to 10.5 % and 24%, in different series.^3^,^7^

Glucose, insulin, and C-peptide levels during oral glucose tolerance tests (OGTT) from 36 thalassaemic patients with normal (n=23), impaired (n=6), or diabetic glucose tolerance (n=7) and 32 control subjects were examined. Patients with impaired glucose tolerance presented hyperinsulinemia and delayed peak insulin during OGTT. The C-peptide/insulin ratio was decreased in patients with abnormal glucose tolerance compared to controls. Insulin sensitivity was significantly reduced in patients with impaired glucose tolerance or diabetes compared to controls.^42^

The considerable variation in the occurrence of glycemic abnormalities can be partially explained by the marked differences in the age composition of cohorts, their genetic background, transfusion regimens, degree of chelation and the screening method used.

## Pathogenic Mechanisms

The pathogenesis of glycaemic abnormalities in TM is complex and multifactorial. The initial insult appears to affect iron-mediated insulin resistance rather than defective insulin production; subsequently, pancreatic ß-cell damage and insulin deficiency develop as a result of direct toxic damage by the non-transferrin bound iron to pancreatic ß-cells. Pancreatic islets have an extreme susceptibility to oxidative damage and to low expression of the antioxidant defence system. Moreover, a high expression of divalent metal transporter predisposes further pancreatic islets to greater accumulation of iron than other cells, potentiating the danger of iron-catalyzed oxidative stress.^43^–^47^

These patients are a very heterogeneous group with some individuals exhibiting mainly insulin deficiency and others predominantly insulin resistance. The traditional concept has been that the initial insult is insulin resistance compensated by hyperinsulinemia, related to liver dysfunction (due to iron deposition), that may interfere with insulin’s ability to suppress hepatic glucose uptake.

Also, at the level of the muscle, iron deposits may decrease glucose uptake. With advancing age, persistent insulin resistance along with a progressive reduction in circulating insulin levels (due to declining β-cell function), with a concomitant reduction in insulin sensitivity, aggravates glucose disturbances leading to glucose intolerance and DM. Then, pancreatic damage and insulin deficiency subsequently develop leading to DM.^4^,^48^–^56^

However, this is not always the sequence of events leading to the development of DM. It has been shown that a defect in β-cell insulin secretion can be present early, before the development of glucose intolerance, resulting from the toxic effect of iron deposition in the pancreas.^54^

In addition, impaired liver function, hepatitis C infection, family history of diabetes mellitus and genetic factor(s) and triggered autoimmune response may also play a role.^4^,^53^

## Assessment of Insulin Resistance/Sensitivity

Various indices of insulin resistance/sensitivity using the data from OGTT have been proposed in the last 20 years.

HOMA-IR has been widely utilized for the estimation of IR. It is calculated by multiplying fasting plasma insulin (FPI) by FPG, then dividing by the constant 22.5, i.e. HOMA-IR = (FPI × FPG)/22.5. The spectrum of HOMA-IR indices in populations is ethnic dependent, and specific cutoff values should be established to allow its use in differentiating normal from impaired insulin sensitivity. However, there is significant variability in defining the threshold of HOMA-IR.^57^–^59^

A summary of reports on HOMA-IR cut-off in different adult populations has been reported by Pilar Gayoso-Diz et al.^57^ The threshold value (66th-90th percentile) reported in 9 studies varied from >1.55 to > 3.8, mean 2.31 ± 0.66. In 140 subjects aged 7–16 years the threshold value was 3. 56

Hyperinsulinemic-euglycemic clamp is known to be the “gold standard” for estimating insulin sensitivity. However, its time and financially consuming realization led to a simplified approach to the quantification of insulin sensitivity. In thalassemic subjects insulin sensitivity (ISI-0,120) is evaluated by a relatively new index derived from OGTT, using the fasting (0 min) and 120 min post-oral glucose (OGTT) insulin and glucose concentrations.^56^

### Recommendations

- The homeostatic model assessment (HOMA-IR) is a validated method to measure insulin resistance from fasting glucose and insulin. However, there is a lack of reference values for subjects with thalassemia (II,B)

## Correlation of Abnormalities of Glucose Homeostasis with iron Overload and Chronic Liver Disease

Elevated serum ferritin concentrations and hepatitis C infection have long been considered as important factors associated with the development of abnormal glucose tolerance in patients with TM.^3^–^5^,^24^,^60^,^61^

### A. Pancreas and iron load

The pancreatic ß-cell function is most closely correlated with pancreatic iron (R2*), while insulin resistance is more strongly associated with somatic iron balance indices (serum ferritin, LIC).

Normal pancreas R2* is < 30 Hz: values of 30–100 Hz constitute mild pancreatic siderosis, 100–400 Hz moderate, and values >400 Hz severe siderosis. Both pancreatic and cardiac R2* are correlated with glucose intolerance and diabetes. The presence of detectable cardiac iron is a relatively good predictor of overt diabetes but lacks sensitivity for milder glucose dysregulation.^38^

### B. Chronic liver disease and iron load

Older patients (>25years) with hemoglobinopathies are at high risk of hepatitis C virus (HCV) infection, as they were transfused before the introduction of HCV donor screening. Despite antiviral therapy, liver disease represents an important cause of mortality in these patients. Chronic HCV infection is the leading cause of liver cirrhosis, hepatocellular carcinoma, and metabolic disorders. Insulin resistance is a representative of the metabolic disorders that leads to the development of diabetes and also affects the outcome of antiviral treatment with interferon.^62^–^64^

De Sanctis et al. studied 29 patients with TM who received intensive subcutaneous (SC) chelation with DFO for periods of 6.2 to 8.8 years. All patients had normal oral glucose tolerance tests before SC chelation therapy was introduced and 22 of 29 patients had normal liver function tests. At the end of the intensive chelation period 12 patients still had normal oral glucose tolerance (7 with normal liver function and 5 with chronic active hepatitis), 11 patients developed impaired glucose tolerance tests (3 patients had normal liver function, 5 had chronic active hepatitis and 3 had cirrhosis), and 6 patients developed frank DM (one with chronic active hepatitis and 5 with cirrhosis).^61^

## Risk Factors Associated with FPG, IGT, and DM

Elevated fasting plasma glucose (FPG) is considered an index of a pre-diabetic state. However, the rate of progression from abnormal FPG to overt diabetes in TM patients is not known.^41^

Risk factors associated with IGT were male sex, poor compliance and/or inefficient dose of chelator(s), increased liver iron concentration (above 7 mg/g dry weight), splenectomy and lower insulin secretion (area under curve) after OGTT, and all factors contributing to high transfusional iron accumulation.^3^,^7^,^63^

The main risk factors associated with DM in addition to the above are advanced age at the start of chelation therapy and liver cirrhosis or severe fibrosis. In some studies, the strongest predictor for the development of diabetes was the duration of transfusion therapy and inefficiency of chelation, with every decade of transfusion exposure increasing the odds of developing diabetes by 2.5 times.^3^,^7^,^65^

Zinc deficiency might lead to a suppression of the ability of the pancreas to secrete sufficient amounts of insulin in response to oral glucose load in patients with TM. Serum zinc levels should be monitored to possibly provide useful complementary information regarding glucose metabolism.^66^,^67^

### Recommendations

- Serum zinc levels should be routinely monitored in these patients so as to provide additional valuable information regarding glucose metabolism. Zinc levels should be measured every 6 months according to the TIF 2014 guidelines. (I,B)

## Natural History of Glycometabolic Status and Risk Factors in TM

The natural history of the glycometabolic state in TM adults is characterized by a deterioration of glucose tolerance (GT) over time.^68^,^69^

Messina et al. studied the evolution of GT, insulin secretion, and peripheral insulin sensitivity during a 3-yr follow-up in a homogeneous population consisting of fourteen non-diabetic adults with TM. GT deterioration over time was accompanied by a reduction of insulin sensitivity, with no concomitant change in insulin secretion. No patient developed diabetes mellitus (DM) during follow-up.^70^

Kattamis et al. reported that the prevalence of IGT increased progressively from 13.4% to 39% over the first 4 years of observation but remained constant during the following 6 years of observation after the intensification of chelation. In contrast, DM had a very low prevalence, beginning with 0.5% at 13–16 years, increasing to 2.4% by the age of 21–24 years.^71^

### Recommendations

- Understanding the sequence of abnormalities in the progression from normal glucose homeostasis to IGT/DM and identifying the risk factors for glycometabolic disturbances in thalassemic patients facilitates the formulation of interventions. (II,B)
- Diagnosis of impaired IFG or IGT indicates a pre-diabetic state which, if not managed appropriately, could progress in TM patients to diabetes (II,B)

## Screening Strategy for Diagnosis of Glucose Abnormalities in Patients with TM

Annual random plasma glucose or fasting plasma glucose measurement as well as the performance of OGTT for all patients with TM aged > 10 years have been used.^3^,^7^

The international guidelines recommend a fasting glucose semi-annually, and if this is greater than 6.1mmol/L, OGTT is indicated. In addition, USA (Standards of Care Guidelines for Thalassemia, 2012) and the ICET-A standards of care 2013 guidelines recommend a two-hour OGTT at 10, 12, 14, and 16 years of age and annually thereafter (Table 1).

Nevertheless, the most accurate method for assessing glucose metabolism in patients with TM is still controversial. Even if the annual OGTT at the age of 10 years is the recommended method, a diagnosis of normal glucose tolerance during OGTT does not exclude abnormal postprandial glucose levels at home. There is now evidence that the OGTT may miss episodes of hyperglycaemia.^39^,^72^,^73^ Noetzli et al. found that fasting glucose > 97 mg/dL and insulin > 9 μU/mL accurately identified an abnormal OGTT result (89% sensitivity, 90% specificity).^38^

Furthermore, some patients with TM and normal fasting and 2-h glucose levels have elevations in the middle of the OGTT (indeterminate glycemia [INDET]) or when assessed randomly or by continuous glucose monitoring.^72^–^75^ The clinical significance of INDET in TM is not known.

The use of continuous glucose monitoring system (CGMS) appears to diagnose early more glycemic abnormalities compared to using HbA1c, fasting glucose and OGTT.^41^,^72^–^75^ The benefit of CGMS, as opposed to other diabetes screening methods, is that it shows a glucose trend, with readings every minute, rather than single-point measurement. This enables capture of increased blood glucose levels over a 24-hour period, which reflects the variable nature of DM.

### Recommendations

- The most accurate method to evaluate altered glucose metabolism in patients with TM is still controversial.(I,A)
- We recommend fasting blood glucose, insulin and calculation of HOMA-IR index.(I,C)
- OGTT in subjects with high serum ferritin can identify patients at high risk of glucose dysregulation and is recommended at 10, 12, 14, and 16 years of age and annually thereafter. (II,B)
- Up to now, little is known about the efficacy of continuous glucose monitoring system (CGMS) as a useful measure for detecting the variability of glucose fluctuations in 24 hours and for assessment of glucose homeostasis in transfusion-dependent beta thalassemia patients, especially due to the lack of clear guidelines. (I,C)
- If a patient with TM develops symptoms of hyperglycaemia (polyuria, polydipsia, weight loss), a blood glucose should be performed.(I,A)

## Clinical Characteristics and Management of IGT and DM in Thalassemia Major

The usual symptoms of polyuria, polydipsia, and weight loss, have been reported to occur in 94.5% of patients with TM and diabetic ketoacidosis (DKA) has been reported to be the presenting manifestation of diabetes in 13.8% to 31.1% of patients.^76^ However, in our personal experience, diabetic ketoacidosis is rare. There was a broad range of symptoms at the clinical onset of diabetes from asymptomatic glycosuria (12 cases) to ketosis (13 cases), or ketoacidosis (four cases). The mean age at diagnosis was 17 years (range 11–24). This may be due to an early detection of mild glucose disturbances.^76^

The mean daily insulin requirement in this series was 0.98 U/kg body weight (range 0.15–1.72). In general terms the metabolic control was good in 4 patients, poor in 8, and very poor in 17. There was a negative correlation between insulin dose and metabolic control. The determination of C-peptide concentrations in 10 patients showed a variation in pancreatic β-cell function: it was increased in one, normal in three, and reduced in 6 cases.^76^ The majority of patients had iron chelation treatment with desferrioxamine on average for 4–9 years.

The onset of diabetes is often associated with the presentation of cardiac dysfunction. Moreover, these patients with clinical diabetes are at a high risk for additional complications such as thyroid dysfunction or hypogonadism and should be strictly monitored.^3^,^4^,^6^,^7^,^76^

Management of DM should be individualised. The first line treatment in all TM patients with glucose disturbances should be an intensification of iron chelation therapy to achieve a negative iron balance. Platis et al. obtained a reversal in one-third of glucose metabolism disorder cases by using combination therapy (DFO and DFP).^77^ Intensive iron chelation therapy with DFO plus DFP seems to be associated with an improvement in glucose intolerance in terms of glucose and insulin secretion, particularly in patients in early stages of glucose intolerance.^24^ Christoforidis et al. showed that patients receiving combined therapy (DFO plus DFP) had an average reduction of insulin resistance index (IRI), accompanied by an average increase in the ß-cell function index and a slight decrease in the insulin sensitivity index (ISI 0–120). In contrast, patients receiving monotherapy either with DFO or DFP showed deterioration in glucose tolerance, indicated by an average reduction of ß-cell function index, a concomitant increase in average IRI and a reduction of ISI 0–120.^78^

There is very limited published data on the efficacy and safety of oral antidiabetic agents in patients with TM. The only drugs used in small studies in this context with good effect were metformin, glibenclamide, and acarbose.^79^–^82^

In established diabetes, the medical treatment depends on the severity of β-cell damage and subsequent insulin deficiency. Introducing oral hypoglycemic drugs in the early stage of diabetes before dependence on insulin proved beneficial in preliminary studies.

Metformin is considered first choice in patients with type 2 diabetes. There is little research in thalassaemia except on one case report in a 25-year-old Tunisian patient.

Insulin resistance also plays a part in the pathogenesis of diabetes in thalassaemia. Since metformin reduces insulin resistance, it could be promising and indeed can be considered in early stages.^79^

The efficacy of glibenclamide administration in the management of glucose disturbances was evaluated in 33 patients with thalassemia, aged 12–30 years (mean 17.4 ± 3.7), in whom diet and exercise failed to regulate hyperglycemia. Improvement of OGTT was observed in 73% of TM patients treated with glibenclamide versus 43% of the control group for a mean period of 59 months. Deterioration of OGTT occurred more rapidly (33.7 ± 26.1 vs. 40.7 ± 34.5 mos), and in more patients of the untreated group (57%) than in treated patients (27%). Among treated patients, the effectiveness of oral hypoglycemic agents lasted longer in patients with diabetes (64.1 ± 40.3 mos) than in patients with impaired curves (54.2 ± 31 mos).^80^

Seventeen TM patients with impaired glucose tolerance (IGT) or non-insulin dependent diabetes mellitus (NIDDM) and hyperinsulinism were treated for 12 months with acarbose (100 mg. orally with breakfast, lunch and evening meals). An improvement in glucose tolerance was observed in 2 out of 11 TM patients with IGT and in all TM patients with NIDDM. Acarbose does not appear to improve insulin resistance directly but may have an indirect effect delaying the absorption of glucose of complex carbohydrates and disaccharides.^81^,^82^

Overall there is limited data on the effect of oral antidiabetic drugs in thalassaemia.

Compared to the general diabetic population, there is no marked difference in the monitoring of glycaemic control in thalassaemic patients. When overt DM develops, patients require daily subcutaneous injections of insulin to normalise blood sugar levels. Since treatment of diabetes in patients with TM is an additional burden, support from doctors and psychologists is needed. Typically, a basal/bolus dose or a combination of both is used to treat DM. Short acting rapid insulin before meals remains the insulin of choice for those without fasting hyperglycaemia.

Self-monitoring of blood glucose (SMBG) is recommended, at least three times a day in patients on insulin therapy. The use of carbohydrate counting and insulin-to-carbohydrate ratios in conjunction with the usual diet to guide insulin therapy can help to optimize glycemic control. Exercise is beneficial and is known to play a vital role in overall health.^4^

Overall, TM patients with diabetes should strive to attain plasma glucose goals as per the ADA recommendations for people with diabetes.^4^,^7^,^83^,^84^

All patients with DM should regularly be monitored for the development of complications. Kidney function and imaging of the fundi should be carried out to evaluate the presence and degree of diabetic complications. However, the incidence of retinopathy and nephropathy in patients with diabetes and thalassaemia is lower than in patients affected by juvenile diabetes.^4^,^7^ This may be due to normal or below normal serum levels of cholesterol and triglycerides, to the frequent presence of hypogonadism and low insulin growth factor 1 (IGF-1)^85^,^86^ as well as comparable shorter period of observation. With regard to macrovascular complications of diabetes, they include ischaemic heart disease, cerebrovascular disease, and peripheral vascular disease. A recent study by Pepe et al. showed that DM in patients with TM significantly increases the risk for cardiac complications, heart failure, hyperkinetic arrhythmias and myocardial fibrosis.^6^

The credibility of Hb A1c as a gold standard for the measurement of control of diabetes in TM patients has been questioned because the hemoglobin composition of patients’ erythrocytes is considerably modified, due to regular and frequent transfusions. As a rule, the patient’s erythrocytes are a mixture of transfused red cells from donors with a normal Hb composition, with Hb A of around 95%, and Hb F of 2–3%. Storage erythrocytes have functional and metabolic differences as well as a considerably shorter life span compared to healthy red cells.^3^,^4^,^74^,^87^–^89^ On the other hand, the results of a recent study showed that assessment of HbA1c prior to transfusion is a reliable index of the average glucose concentration for the period between transfusions ranging from 2–4 weeks and up to 40 days. In the Kattamis et al. study a cut off value of 6.8–7% was suggestive of diabetes and values between 6 and 7% of prediabetes.^90^ Further studies are needed to confirm these observations.

Serum fructosamine levels have been proposed as an appropriate laboratory measurement when monitoring long-term glycemic control in patients with TM and diabetes mellitus.^4^,^7^ A single measurement with this assay provides an assessment of glycemic control over the preceding 2–3 weeks. Some limitations with the use of the fructosamine assay have been noted, including the short half-life of fructosamine which might result in fructosamine being more susceptible to rapid changes in blood glucose, and difficulty with standardization of the assay because albumin can be profoundly affected by disease states and drugs.^74^,^91^

Diabetes in pregnancy is associated with risks to the woman and to the developing foetus. The International clinical guideline contains recommendations for the management of diabetes and its complications in women who wish to conceive and those who are already pregnant.^90^,^91^

Women with TM are potentially at high risk for development of hyperglycemia during pregnancy (gestational diabetes mellitus). Therefore, those who are contemplating pregnancy should be evaluated prior to conception to rule out any impairment of glucose homeostasis. Specific criteria for the diagnosis of gestational diabetes should be used.^92^,^93^

### Recommendations

- Intensive iron-chelation therapy and prevention and treatment of chronic hepatitis C are now the most important issues in managing impairment of glucose homeostasis in patients with transfusion dependent β-thalassemia. (II,A)
- Management of DM should be individualised.(II,C)
- During initiation of insulin, blood glucose monitoring both pre- and post-prandially as well as at bedtime and overnight may help to determine dosage requirements.(II,A)
- Patients with diabetes who are on insulin should perform self-monitored blood glucose testing at least three times a day.(II,A)
- Continuous glucose monitoring (CGMS) is under investigation as a potential new measure of prandial glucose control, especially in the more difficult cases. (II,A)
- Patients with TM should strive to attain plasma glucose goals as per the ADA recommendations for all people with diabetes.(I,A)
- There is limited published data on the efficacy and safety of oral antidiabetic agents. (II,A)
- Glycated hemoglobin A1c reflects a mean glycemia over the preceding 3 months (erythrocyte life span). In diabetes management, the target value is set below 6.5%, to reduce the risk of chronic complications. However, HbA1c is a poor marker in subjects with diabetes and hemoglopinopathies.(I,A) Fructosamine determination is useful for monitoring diabetes in these patients.(I,A)
- TM women with normal glucose tolerance pre-pregnancy should still be advised that they may develop glucose intolerance later in pregnancy, and that repeat OGTT should be performed at both 12–16 and 24–28 weeks gestation with measures at 0, 1 and 2 h using the specific gestational diabetes criteria.(I,A)
- Women with diabetes who are planning to become pregnant should be informed that establishing good glycaemic control before conception and continuing this throughout pregnancy will reduce the risk of miscarriage, congenital malformation, stillbirth and neonatal death. It is important to explain that risks can be reduced but not eliminated. (I,A)
- TM women with pre-existing diabetes should have pre-pregnancy counselling and planning to aim for optimal glycemic control before and throughout pregnancy to minimize adverse pregnancy outcomes. (I,A)
- All pregnant patients with DM should regularly be monitored for the development of complications. (I,A)
- Plasma glucose levels should be monitored closely during the peri-partum period and until hospital discharge. (II,C)
- Chelation treatment should be interrupted during pregnancy.(I,C)
- Diabetic patients with TM should regularly be seen by a specialized multidisciplinary team with expertise in both diabetes and TM, including ongoing diabetes self-management education.(I,A) The team should include an endocrinologist and dietician with experience in TM. (I,C)

## Figures and Tables

**Table 1:** The international recommendations for the screening of altered glucose homeostasis in transfusion depended on thalassemia major.

References	Recommendations
TIF (Guidelines for the Management of Transfusion Dependent Thalassaemia (TDT), 3rd edition, 2014)	OGTT: Q 1 y starting at puberty and FBG every 3 months
USA (Standards of Care Guidelines for Thalassemia, 2012)	FPG: Q 6 mo starting at 5 y OGTT: at 10, 12, 14, 16 y then Q 1 y or as indicated by FPG If FPG is greater than 110 mg/dl an oral OGTT is indicated
Canada (Guidelines for the Clinical Care of Patients with Thalassemia in Canada, 2009)	FPG: Q 6 mo starting at puberty
UK (Standards for the Clinical Care of Children and Adults with Thalassaemia in the UK, 2008)	FPG: Q 3–6 mo starting at puberty or starting at 10 y if positive family history OGTT: Q 1 y starting at puberty or starting at 10 y if positive family history
Australia (Int Med J 2010 ; 40:689–96)	Yearly fasting blood glucose after puberty Proceed to 75g OGTT if indicated
Malaysia (Management of transfusion dependent thalassaemia, 2009)	FPG or a 2 hour OGTT should be performed annually on thalassaemia patients > 10 years old.
I-CET (Indian J Endocrinol Metab 2013;17:8–18)	FPG annually from the age of 5 years. A 2-h OGTT, preferably combined with insulin secretion determination, should be performed at 10, 12, 14, and 16 years of age and annually thereafter. If fasting serum glucose is >110 mg/dl, OGTT is indicated independently of patient’s age

Legend: TIF: Thalassaemia International Federation; Q: every; mo: month; y: year; I-CET: International Network of Clinicians for Endocrinopathies in Thalassemia; FPG: Fasting Plasma Glucose; OGTT: Oral Glucose Tolerance Test.
